# Beyond the Canonical Endocannabinoid System. A Screening of PPAR Ligands as FAAH Inhibitors

**DOI:** 10.3390/ijms21197026

**Published:** 2020-09-24

**Authors:** Leonardo Brunetti, Antonio Carrieri, Luca Piemontese, Paolo Tortorella, Fulvio Loiodice, Antonio Laghezza

**Affiliations:** Department of Pharmacy and Pharmaceutical Sciences, University of Bari “A. Moro”, via E. Orabona 4, 70125 Bari, Italy; l.brunetti2@studenti.uniba.it (L.B.); antonio.carrieri@uniba.it (A.C.); luca.piemontese@uniba.it (L.P.); paolo.tortorella@uniba.it (P.T.)

**Keywords:** multitarget, anandamide, FAAH, PPARs, Alzheimer’s disease, endocannabinoids, inflammation

## Abstract

In recent years, Peroxisome Proliferator-Activated Receptors (PPARs) have been connected to the endocannabinoid system. These nuclear receptors indeed mediate the effects of anandamide and similar substances such as oleoyl-ethanolamide and palmitoyl-ethanolamide. An increasing body of literature describing the interactions between the endocannabinoid system and PPARs has slowly but surely been accumulating over the past decade, and a multitarget approach involving these receptors and endocannabinoid degrading enzyme FAAH has been proposed for the treatment of inflammatory states, cancer, and Alzheimer’s disease. The lack of knowledge about compounds endowed with such an activity profile therefore led us to investigate a library of readily available, well-characterized PPAR agonists that we had synthesized over the years in order to find a plausible lead compound for further development. Moreover, we propose a rationalization of our results via a docking study, which sheds some light on the binding mode of these PPAR agonists to FAAH and opens the way for further research in this field.

## 1. Introduction

Peroxisome proliferator-activated receptors (PPARs) are a family of nuclear receptors which have been a useful therapeutic target for the treatment of metabolic disorders comprising obesity, type 2 diabetes mellitus (T2DM), dyslipidemia, and hypertension [1,2,3]. This family includes three receptor subtypes, namely PPARα, PPARγ, and PPARδ (or β/δ) which differ for their tissue distribution, ligand specificity, and also for the downstream effects of their activation. However, they are all involved in lipid and carbohydrate homeostasis, and can be regarded as “master modulators” of energetic metabolism. Recent studies have demonstrated that the full activation of these receptors, and in particular of PPARγ, which is the target of the currently used antidiabetic drugs thiazolidinediones, is associated with unwanted effects. However, recent observations indicate that an appropriate modulation of PPAR activity, achievable by the so-called selective PPAR modulators (SPPARMs), appears the key challenge to uncouple the adverse effects of a ligand from its therapeutic effects. These studies, therefore, pave the way to a new and hopeful perspective for treatment of T2DM and dyslipidemia [4]. An important characteristic of PPARs is the lack of an actual class of endogenous ligands. Indeed, each receptor subtype is bound, with relatively low affinity, by a plethora of compounds (either endogenous or food-derived) [1]. This promiscuity implies that PPARs share some mediators with other signaling systems; one of these is the endocannabinoid system (ECS) [5]. The ECS comprises the canonical G-protein coupled cannabinoid receptors (CBRs) CB1 and CB2 and the endocannabinoid substances arachidonoyl-ethanolamide (AEA, or anandamide) and 2-arachidonoyl-glycerol (2-AG). The ECS is also affected by other substances, structurally similar to AEA, belonging to the class of N-acyl-ethanolamines (NAEs), the most important of which are oleoyl-ethanolamide (OEA) and palmitoyl-ethanolamide (PEA). Although they do not directly act on CBRs, they are capable of mediating their activation via the so-called “entourage effect”, which is caused by these mediators’ ability to compete with endocannabinoids for their catabolic enzymes, such as fatty acid amide hydrolase (FAAH) [5,6,7].

FAAH is a serine hydrolase which is physiologically found as a dimer bound to the endoplasmic reticulum (ER) that, along with other enzymes such as N-acylethanolamine acid amidase (NAAA), quickly and completely hydrolyzes AEA and other NAEs. It is important to note that, among NAEs, AEA, OEA, and PEA all act as PPARα agonists, while only AEA is also capable of acting as a PPARγ agonist [5,6,7].

The pharmacological inhibition of FAAH results in an enhancement of the endocannabinoid tone, which has many potential advantages as a therapeutic strategy, since the ECS is essential to many physiological processes in the central nervous system (CNS) and is usually upregulated as a protective response to various pathological conditions, such as pain, inflammation and the expansion of neoplastic clones. Therefore, a pharmacological enhancement of the endocannabinoid tone could be useful to treat conditions which are characterized by such events [8,9]. Moreover, this pharmacological strategy does not result in the typical collateral psychoactive effects mediated by exogenous phytocannabinoids such as Δ^9^-tetrahydrocannabinol, including sedation, hyperphagia, and hypomotility. FAAH inhibitors in particular do not induce tolerance and have very few toxic effects [10,11,12]. The overlap between endocannabinoid and PPAR signaling suggests that it is possible to expand the activity profile of PPAR ligands by coupling their activity with the inhibition of enzymes such as FAAH. The applications of such an activity profile would be many, including the treatment of cancer [13], of neurodegenerative diseases [14], and of alcohol withdrawal [15], other than metabolic syndrome. In this particular case, the anti-inflammatory effects linked to endocannabinoid activation would be particularly helpful in reducing the hyperinflammatory state of metabolic syndrome patients; it is however important to note that PPARs themselves are among the most important mediators of such effects [16,17,18,19]. Moreover, ligands of this kind could help patients comply with the lifestyle changes that are the first steps in the therapy of metabolic syndrome, such as reducing the consumption of alcoholic beverages and tobacco smoke. Indeed, the activation of PPARα mediated by a stable analog of OEA could alleviate the symptoms of nicotine abstinence and reduce the reward mechanisms linked to its consumption, while PPARγ activation can reduce the reward mechanisms linked to alcohol consumption [15]. Unfortunately, however useful this activity profile may seem to be, no molecules have been developed to date for this kind of application. Therefore, in order to obtain compounds capable of activating PPARs and inhibiting FAAH, we started with a preliminary investigation of the FAAH inhibitory activity of a number of aryloxyacetic PPAR agonists, both known in the literature and synthesized in our laboratory. This approach was further encouraged by the structural similarities between the aryloxyacetic class of PPAR agonists, whose activity has been widely studied in the past two decades [20,21,22,23], and the arylacetic class of cyclooxygenase (COX) inhibitors, which were recently shown to be moderately active on FAAH [24,25,26].

Natural compounds were also tested with the aim of finding structural elements which could prove useful to further drug design. Moreover, we report the synthesis of novel derivatives of rosmarinic acid, a natural compound, and their biological activities on FAAH and PPARs. The synthesis of as yet unpublished aryloxyacetic derivatives with limited activity as FAAH inhibitors is also reported. After the screening, docking studies were conducted for the most interesting compounds on FAAH, in order to gain a rational understanding of the measured data, and to help plan further synthesis efforts.

## 2. Results and Discussion

### 2.1. Screening Results

**Figure 1:**
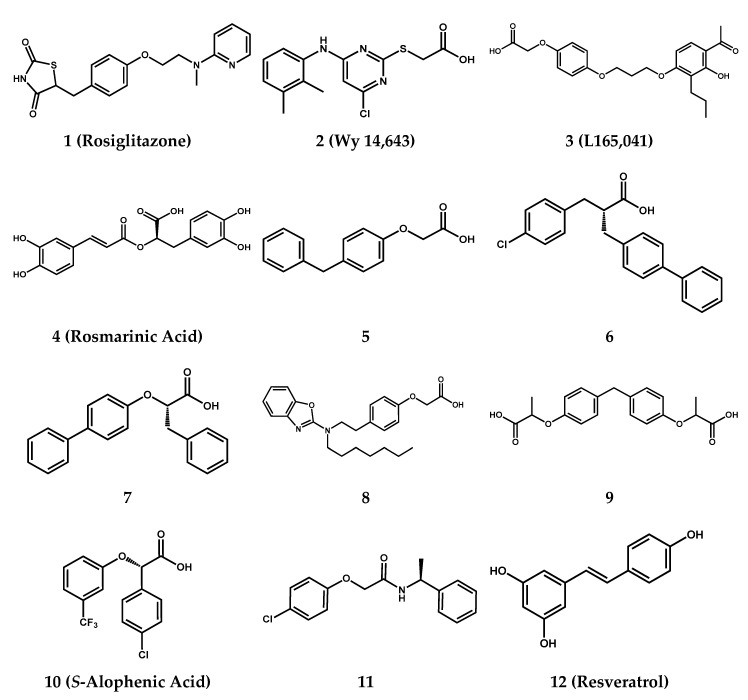
Molecular structures of the first 12 compounds involved in the screening.

At first, a library of 12 compounds (reported in Figure 1), comprising mostly synthetic PPAR agonists and two natural compounds, was tested for FAAH inhibition. The first three compounds are standard PPAR agonists, whereas **4**–**12** were selected on the basis of their well-known multi-target in vitro activity as well as their specific cellular and/or in vivo effects [27,28,29,30].

While most of these compounds showed no relevant activity towards FAAH, some of them showed promising results; these were compounds **4**, **5**, **8**, and **12** (resveratrol). In particular, this last compound can interact with PPARγ, albeit as an antagonist [27] and has numerous other reported biological activities [28]. It is worth noting that compounds **1** (Rosiglitazone), **2** (Wy 14,643), and **3** (L165,041) showed little to no activity on FAAH, demonstrating that a more specific drug design process is necessary for the obtainment of dual-acting compounds. These biological results are reported in Table 1.

We subsequently focused our attention on compounds **5** and **8**, which are PPAR agonists of the phenoxyacetic class, showing an interesting activity profile. In particular, **5** showed a balanced activity profile, with comparable potency values towards all tested targets, even though its efficacy towards PPARs proved to be very low. The ureidofibrate **8** showed instead much higher potencies and efficacies towards PPARα and PPARγ, while keeping an IC_50_ value towards FAAH that was quite similar to that of compound **5**. 

Many relevant structural modifications were introduced to the main aryloxyacetic scaffold of compound **5**. One of the main points of structural variability of these analogues (**13**–**21**) is the carbon in alpha to the carboxylic group, which can be substituted with a methyl or a benzyl moiety. The aromatic “tail” of these compounds is another important site of structural variations—mainly, the linker between the two aromatic rings can be lengthened, or substituted, either with the introduction of heteroatoms, or with the introduction of a more rigid planar system as seen with compounds **18** and **19**. In the case of compound **8**, the only modifications concerned the carbon in alpha to the carboxylic group, in which one or two methyls or a methyl alongside an ethyl were introduced, while the 2-(N-heptylamino)benzoxazole moiety was left unchanged (as seen with compounds **22**–**26**). The general rule that emerges from their activity data (shown in Table 2) is that steric hindrance on the alpha carbon strongly reduces potency towards FAAH, skewing the activity profile of more hindered molecules towards PPAR agonism. With the phenoxy moiety being equal, this feature of phenoxyacetic acids can be seen comparing compounds **13** and **14**:**13**, with only a methyl group on its alpha carbon, is significatively less active than its unsubstituted analogue **5**, while **14**, with a bulky benzyl group, is completely inactive towards FAAH. Similarly, **16** is less active on FAAH than its unsubstituted analogue **15** and ureidofibrates **22**–**26** are all less active than **8**.

However, compound **18** is an interesting exception to this rule, being an FAAH inhibitor in the high micromolar range and a partial agonist of PPARα and γ in the low micromolar range. When compared to its cis- geometric isomer **19**, compound **18** is substantially more active as a FAAH inhibitor, which allows to suppose that the presence of a planar system such as a trans-stilbenic moiety could be relevant for FAAH inhibitory activity, as seen for resveratrol as well. 

Only few optically pure compounds of this class have been tested as FAAH inhibitors and did not show any particular stereoselectivity toward this target. However, the low activity of these molecules makes it difficult to determine with certainty such a lack of stereoselectivity which will be object of further studies. 

Rosmarinic acid (**4**) was reported in the literature as a PPARγ agonist [30] and presents some structural similarity to arylacetic acids. Although we did not find any activity of this compound on either PPARα or PPARγ, in light of its positive result as an inhibitor of FAAH, we decided to synthesize a short series of new derivatives of this compound and its natural analogue clovamide (**27**) (Table 3) by which we evaluated the relevance of the phenolic groups and the double bond. The former were removed or methylated, whereas the latter was reduced or incorporated in a benzene ring. These derivatives were subsequently tested on PPARα, PPARγ, and FAAH to verify if any of them was endowed with the desired activity profile.

Unfortunately, only compounds **(S)-28** and **33** were found to be active on either FAAH or PPARs (Table 4). The presence of catecholic OH- groups in the cinnamic moiety of these compounds seems to be essential for FAAH inhibition, but completely abolishes any kind of agonist activity on PPARs. Given the mutual exclusivity of the structural requirements of this class of compounds, we decided to set them aside and did not consider them for further investigation. 

### 2.2. Chemistry

**Scheme 1:**
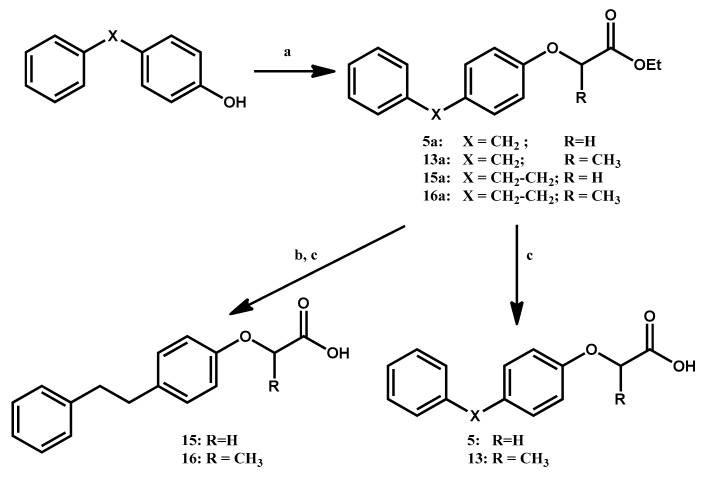
Synthesis of aryloxyacetic analogues: (a) ethyl 2-bromopropanoate or ethyl bromoacetate, NaH, DMF_dry_, N_2_, 0 °C > 65 °C; (b) H_2_, Wilkinson’s catalyst, THF/EtOH, RT; (c) NaOH 2N, THF, RT.

The aryloxyacetic analogues **5**, **13**, **15,** and **16** were synthesized starting from the appropriate phenol (4-hydroxy-diphenylmethane for compounds **5** and **13,** 4-hydroxy-stilbene for compounds **15** and **16**), which was reacted with ethyl 2-bromopropanoate or ethyl bromoacetate in the presence of sodium hydride in anhydrous DMF, giving intermediates **5a**, **13a**, **15a,** and **16a**. Then, **5a** and **13a** were directly hydrolyzed in NaOH/THF to the desired compounds **5** and **13**, while **15a** and **16a** were first reduced via catalytic hydrogenation and then hydrolyzed, giving compounds **15** and **16** (Scheme 1).

**Scheme 2:**
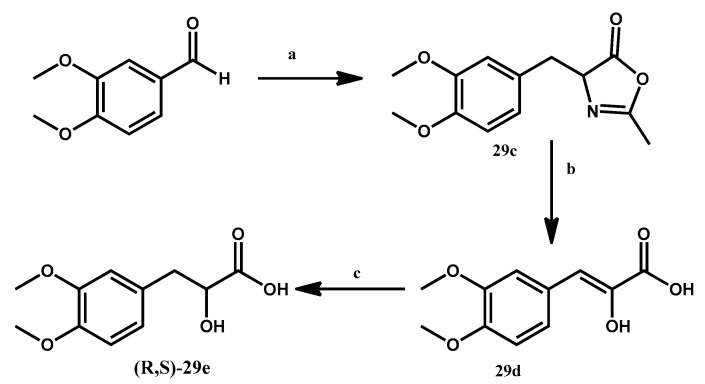
Synthesis of the intermediate **29e**. (a) N-acetylglycine, NaOAc, Ac_2_O, 18 h, 110 °C; (b) HCl 3M, 6 h, 100 °C; (c) MeOH (25%), NaOH 0.1M, NaBH_4_, 17 h, RT.

Rosmarinic acid derivatives **28**–**30** were synthesized starting from an appropriately substituted phenyllactic acid. While both (*R*)- and (*S*)-phenyllactic acids are commercially available, 3,4-dimethoxyphenyllactic acid (shown as intermediate **29e**) had to be synthesized. To this end, 3,4-dimethoxybenzaldehyde was reacted with N-acetylglycine in the presence of sodium acetate and acetic anhydride, obtaining oxazolone intermediate **29c**, which was then hydrolyzed with HCl 3N to give the enol **29d**. This was in turn reduced with NaBH_4_ in MeOH/NaOH at room temperature to obtain the intermediate **29e** (Scheme 2). 

**Scheme 3:**
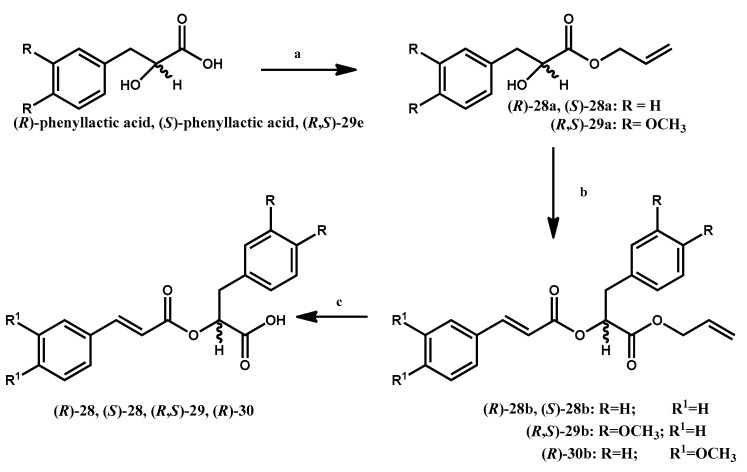
Synthesis of rosmarinic acid derivatives **28**–**30**. (a) Allyl Alcohol, p-TsOH, 11 h, 100 °C; (b) DMAP, EDCI, Trans-cinnamic acid or 3,4-dimethoxycinnamic acid, 24 h, RT; (c) Pd(PPh_3_)_4_, morpholine, 2 h, RT.

Afterward, the appropriate phenyllactic acids were condensed with allyl alcohol in the presence of tosylic acid at 100 °C; then, their hydroxy group was esterified with cinnamic acid, obtaining intermediates **(R)-28b**, **(S)-28b**, **(R)-30b**, or 3,4-dimethoxycinnamic acid, obtaining intermediate **(R)-29b**. The allyl esters were subsequently hydrolyzed using tetrakis(triphenylphosphine)palladium in morpholine obtaining the desired acids (Scheme 3).

**Scheme 4:**
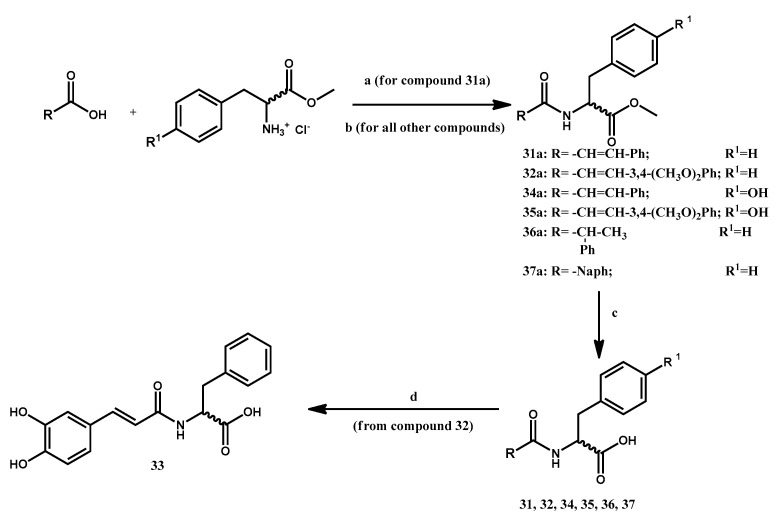
Synthesis of clovamide derivatives **31**–**37**. (a) DCC, HOBt x H_2_O, TEA, THF/CHCl_3_, 0 °C→RT; (b) EDCI, HOBt x H_2_O, N-Methylmorpholine, DMF/CH_2_Cl_2_, 0 °C→RT; (c) LiOH x H_2_O, THF/H_2_O, 0 °C→RT; (d) BBr_3_ 1M, CH_2_Cl_2_.

Compounds **31**–**37**, derivatives of natural compound Clovamide, were synthesized starting from an appropriate cinnamic acid which was condensed with phenylalanine-methylester hydrochloride, (S)-phenylalanine or (S)-tyrosine by using DCC and HOBt in THF/CHCl_3_ or EDCI and HOBt in DMF/CH_2_Cl_2_ as condensing agents. Intermediates **31a**, **32a**, and **33a**–**37a** were subsequently hydrolyzed with LiOH in THF/H_2_O, affording the corresponding desired acids **31**, **32**, and **33**–**37**. Of these, compound **32** was demethylated with boron tribromide in dichloromethane, resulting in compound **33** (Scheme 4).

### 2.3. Molecular Modeling

To gain fresh insight into the binding modes and biological activities of the compounds previously discussed, we carried out a docking study on some compounds selected as representative of the dataset. Although many of the studied derivatives show a significant PPAR agonist profile, we preferred to focus our attention on FAAH inhibition, since numerous and ample structure-based perceptions on the binding mode of phenoxyacetic acids to PPARs have already been widely accomplished by our research group ([25] and references therein). Hence, the X-ray crystal structure of recombinant rat FAAH in complex with carprofen, a non-steroidal anti-inflammatory drug also endowed with some activity as an inhibitor of this enzyme (IC_50_ = 79 ± 20 μM), was then used as a blueprint of sorts for our docking studies. Although the biological assays were carried out on human recombinant FAAH, these two variants of this enzyme share more than 90% of their sequence [26].

Crystallographic data depict the FAAH catalytic region as a membrane-accessing tunnel followed by an elongated cavity where the enzyme’s active site is located. Indeed, in the non-covalent ligand/enzyme complex, carprofen is merged in the aforementioned cleft, anchoring the partially solvated propanoic acid group, through one direct H-bond with W531 indole ring, mediating additional interactions most likely involving R486 and T488 side-chains and with the help of water molecules. Conversely, the heterocyclic moiety and halogen atom are deeply buried in a relatively sized and lipophilic gorge comprising hydrophobic amino-acid residues, namely L192, F381, F432, M436, and I149. Therefore, taking the FAAH-Carprofen complex as reference, we decided to sample the complementarity between the FAAH binding site and some selected representative compounds from our dataset, in light of relevant clues from the experimentally obtained IC_50_ data. 

**Figure 3:**
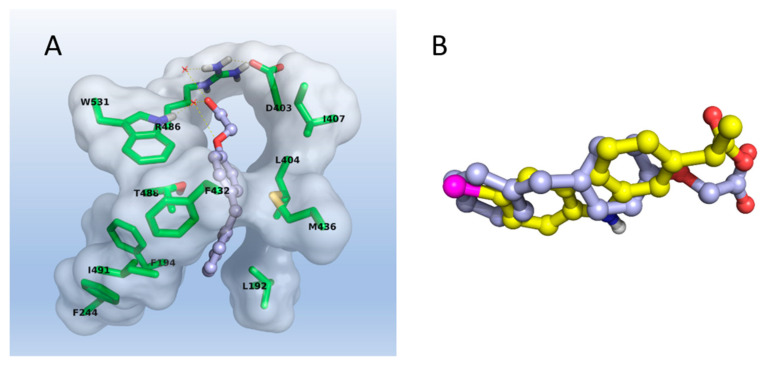
(**A**): docking pose of compound **5**. (**B**): molecular superposition with carprofen (yellow carbons).

To this end, compound **5** was first tested, and subsequently docked to the FAAH binding site, comparing the achieved pose to X-ray data. It is very interesting to note how the phenoxyacetic derivative presents an evident structural relation to carprofen, as proved by a more than acceptable Tanimoto molecular similarity coefficient (0.605 according to ROCS shape-based matching algorithm) [33,34]. Furthermore, some common pharmacophoric elements can be appreciated: an ionized carboxy group, acting as an ionic binding lock, is in fact present in both molecules, while the aromatic benzyl moiety, stabilizing the binding due to favourable hydrophobic contacts involving L192, M436, L404, F432 and I491 side chains, slightly recalls the carbazolic ring of carprofen (see Figure 3). The presence of two water molecules assisting ligand binding to the enzyme surface more widely exposed to solvent can also be observed. The overall goodness of the accomplished pose is indeed confirmed by a favourable (>−7.0 kcal/mol) free energy of binding, ligand efficacy (~−0.4) and significant contact surface area (see Table 5).

**Figure 4:**
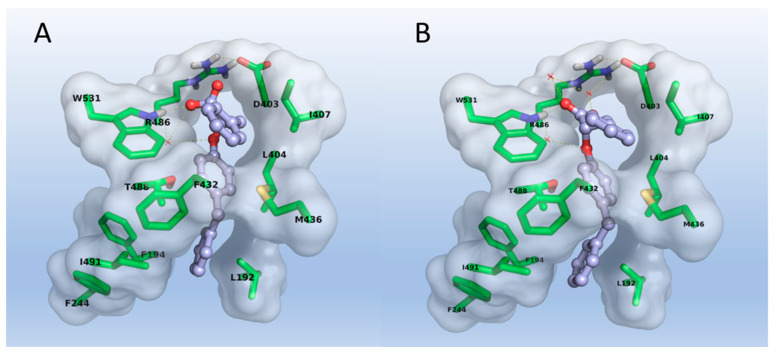
Docking poses of (*R*), (**A**), and (*S*), (**B**) enantiomer of compound **14** into the FAAH binding site.

Docking of compound **14** might at least in part explain its complete lack of FAAH activity, that could be indeed ascribed to the steric hindrance occurring at the alpha-carbon in both (*R*) and (*S*) configurations, the benzyl fragment being a probable chemical cliché able to hamper the correct insertion in the deeper part of the binding site of the whole molecular scaffold, as can be appreciated in Figure 4. This evidence, however, cannot be sufficient to justify the total inactivity of compound **14** towards FAAH, which might be perhaps due to additional molecular descriptors as suggested by a large (>2.5 log units) difference in terms of lipophilicity as suggested by the measured LogD values (0.27 and 2.62 for compound **5** and **14** respectively).

**Figure 5:**
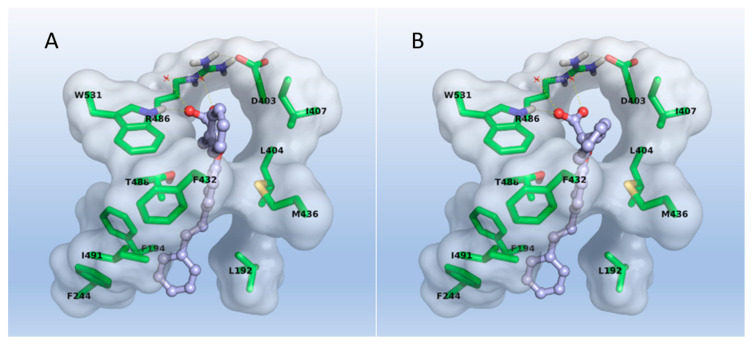
Docking poses of (*R*), (**A**), and (*S*), (**B**) enantiomer of compound **18** into the FAAH binding site.

On the other hand, the most active compound **18** better accomplishes the structural requirements of FAAH, thanks to its lipophilic, flat, and rigid trans-stilbenic tail. This fragment can more tightly nail (see Figure 5) the inhibitor on the membrane-accessing tunnel, allowing a tighter binding, and higher (CSA > 480 Å^2^) contacts on the protein-ligand interface with respect to the inactive derivative. Indeed, the same moiety accomplishes intense π-π stackings occurring in an additional lateral pocket involving F432, F194, and F244 where the aromatic pendent can be easily accommodated, in contrast to what occurs for compound **14** lacking such structural motif.

Interestingly, docking also suggested that stereoselectivity is not an intrinsic feature of FAAH, as confirmed by similar topology and comparable better free energy of binding values in both the (*R*) and (*S*) configurations of compound **18** enantiomers.

To a lesser extent, a similar binding mode was also achieved for the ureidofibrate compound **8** (see Supplementary Figure S1).

## 3. Materials and Methods

### 3.1. Chemical Methods

Melting points were determined in open capillaries on a Gallenkamp electrothermal apparatus and are uncorrected. Mass spectra were recorded on a HP MS 6890-5973 MSD spectrometer, electron impact 70 eV, equipped with a HP ChemStation or with an Agilent LC–MS 1100 Series LC–MSD Trap System VL spectrometer, electrospray ionization (ESI). ^1^H-NMR spectra were recorded using the suitable deuterated solvent on a Varian Mercury 300 NMR Spectrometer or with an Agilent VNMRS500. Chemical shifts (δ) are expressed as parts per million (ppm) and the coupling constants (J) in Hertz (Hz). Microanalyses of solid compounds were carried out with a Eurovector Euro EA 3000 model analyzer. The analytical results are within ±0.4% of theoretical values. Column chromatography was performed using Geduran silica gel 60 A° (63–200 µm) as a stationary phase. Optical rotations were determined with a Perkin-Elmer 341 polarimeter at room temperature (20 °C). Concentrations are expressed as g/100mL. Chemicals were purchased from Aldrich Chemicals (Milan, Italy) and were used without any further purification. Spectral analysis data (GC-MS, HRMS and NMR) can be found in the Supplementary Information.

#### 3.1.1. General Procedure for the Preparation of Ethyl Aryloxyacetates **5a**, **(R,S)-15a** and Ethyl 2-Aryloxy-propanoates **(R,S)-13a**, **(R,S)-16a**

At T = 0 °C, using anhydrous DMF as solvent, NaH is mixed with the appropriate phenol (either 4-benzylphenol or 4-hydroxy-trans-stilbene) in a 5:1 stoichiometric ratio. After 30 min, the resulting suspension is brought to room temperature. A DMF solution of ethyl 2-bromoacetate or ethyl 2-bromopropanoate is subsequently added dropwise in a 2:1 stoichiometric ratio with the phenol. The mixture is stirred at 65 °C for 2–6 h, then the solvent is evaporated under vacuum. The residue is dissolved in EtOAc, washed once with NH_4_Cl, once with NaOH 0.5N, and once with NaCl_ss_. The organic phase is dried over Na_2_SO_4_, filtered over cotton and the solvent is evaporated under vacuum. The crude product is then purified via column chromatography with eluent Hex/EtOAc 95:5.

**Ethyl 4-benzyl-phenoxyacetate 5a**

61% yield.

**(*R*,*S*)-Ethyl 2-(4-benzylphenoxy)-propanoate 13a**

83% yield.

**Ethyl trans-(4-styryl-phenoxy)-acetate 15a**

73% yield.

**(*R*,*S*)-Ethyl 2-trans-(4-styryl-phenoxy)-propanoate 16a**

71% yield.

#### 3.1.2. General Procedure for the Preparation of Ethyl (4-Phenethyl-phenoxy)-acetate **15b** and (*R*,*S*)-Ethyl 2-(4-Phenethyl-phenoxy)-propanoate **16b**

A suspension of Wilkinson’s catalyst (0.15 mmol) in absolute EtOH is added to a solution of the appropriate ester 15a or 16a (3 mmol) in THF. The resulting mixture is then placed in an autoclave at 15atm of H2 pressure and stirred for 48 h. Afterwards, the catalyst is removed via filtration over celite and the solvent is evaporated under vacuum. The crude product is then purified via column chromatography over silica gel (eluent Hex/EtOAc 95:5), affording the purified product.

**Ethyl (4-phenethyl-phenoxy)-acetate 15b**

79% yield.

**(*R*,*S*)-Ethyl 2-(4-phenethyl-phenoxy)-acetate 16b**

58% yield.

#### 3.1.3. General Procedure for the Preparation of 4-Aryl-phenoxyacetic Acids **5** and **15** and (*R*,*S*)-2-(4-Arylphenoxy)-propanoic Acids **13** and **16**

The appropriate ester is dissolved in 30 mL THF and mixed with a 10 mL solution of NaOH 6N. The solution is stirred at room temperature for 6–12 h. The solvent is then evaporated under vacuum and the residue is brought to acid pH with HCl 6N. The resulting solution is extracted three times with diethyl ether, and the organic phases are joined, washed once with NaCl_ss_, dried over Na_2_SO_4_, filtered over cotton, and the solvent is evaporated under vacuum. The desired product is obtained via crystallization from CHCl_3_/Hex. 

**4-benzyl-phenoxyacetic acid 5**

56% yield. m.p. 117–118 °C.

**(*R*,*S*)-2-(4-benzyl)-phenoxypropanoic acid 13**

71% yield. m.p. 102–104 °C

**4-phenethyl-phenoxyacetic acid 15**

59% yield. m.p. 130–131 °C

**(*R*,*S*)-2-(4-phenethyl)-phenoxyacetic acid 16**

62% yield. m.p. 106–107 °C

#### 3.1.4. Preparation of 4-[(3,4-Dimethoxyphenyl)methyl-idene]-2-methyl-4,5-dihydro-1,3-oxazol-5-one **29c**

N-Acetylglycine (15 mmol), sodium acetate (15 mmol) and acetic anhydride (60 mmol) were added to 3,4-dimethoxybenzaldehyde (15 mmol). The reaction is stirred for 18 h at 110 °C. Upon cooling, the formation of a yellow solid precipitate is observed. This solid is filtered, then washed with 10 mL of an EtOH/H_2_O 1:1 solution, affording the desired compound as a yellow solid. 25% yield. 

#### 3.1.5. Preparation of 3-(3,4-Dimethoxyphenyl)-2-hydroxyacrylic Acid **29d**

Breifly, 25 mL of HCl 3N are added to compound 15c (3.75 mmol). The reaction is stirred for 6 h at 100 °C. Upon cooling, a red solid is precipitated. The desired compound is crystallized in MeOH/H_2_O. 31% yield.

#### 3.1.6. Preparation of (*R*,*S*)-3-(3,4-Dimethoxyphenyl)-2-hydroxypropanoic Acid **29e**

At T = 0 °C, a 25% solution of MeOH in H_2_O is added to 15d (1.16 mmol), NaOH is slowly added until pH = 10, then NaBH_4_ (1.74 mmol) is added. The reaction is stirred for 17 h at room temperature. The resulting solution is acidified with HCl 1N, then saturated with NaCl, and extracted with EtOAc (5 × 10 mL). The organic phases are joined, dried over anhydrous Na_2_SO_4_ and filtered over cotton, after which the solvent is evaporated in vacuo, affording a light brown oil. This crude product is purified via column chromatography with eluent CHCl_3_/MeOH 10:1, affording a yellow oil. 89% yield.

#### 3.1.7. General Procedure for the Preparation of Allyl 3-Phenyl-2-hydroxypropanoates **(R)-28a**, **(S)-28a**, (*R*,*S*)29a

The appropriate 3-phenyl-2-hydroxypropanoic acid is dissolved in anhydrous toluene and mixed with allyl alcohol and paratoluensulfonic acid in a stoichiometric ratio of 1:1.2:0.2. The resulting mixture is stirred at 100 °C for 6 h. The solvent is removed in vacuo, the crude product is dissolved in EtOAc and washed twice with NaHCO_3ss_ and one with NaCl_ss_. The organic phase is dried over Na_2_SO_4_, filtered over cotton and the solvent is evaporated in vacuo. 

**(*R*)- and (*S*)-Allyl 3-phenyl-2-hydroxypropanoate 28a**

56–89% yield.

**Allyl 3-(3,4-dimethoxyphenyl)-2-hydroxypropanoate 29a**

46% yield. 

#### 3.1.8. General Procedure for the Preparation of 1-(Allyloxy)-1-oxo-3-phenylpropan-2-yl-2-phenylacrylates

The appropriate allyl 3-phenyl-2-hydroxypropanoate (1 mmol), the appropriate cinnamic acid (1.1 mmol), EDCI (1.3 mmol), and DMAP (0.1 mmol) are dissolved in CH_2_Cl_2_ and stirred for 24 h at RT. The resulting mixture is washed once with NH_4_Cl_ss_, diluted with more CH_2_Cl_2_ and washed once with NaCl_ss_. The organic phase is dried over anhydrous Na_2_SO_4_, filtered, and the solvent is evaporated under vacuum, affording a yellow crude oil which is subsequently purified via column chromatography on silica gel (eluent Hex:EtOAc 9:1), yielding the desired product as a yellow oil. 

**(*R*)- and (*S*)-1-(allyloxy)-1-oxo-3-phenylpropan-2-yl-2-phenylacrylate (*R*)-28b**

39–52% yield.

**1-(allyloxy)-1-oxo-3-(3,4-dimethoxyphenyl)-propan-2-yl 2-phenylacrylate 29b**

56% yield.

**(*R*)-1-(allyloxy)-1-oxo-3-phenyl-propan-2-yl-2-(3,4-dimethoxyphenyl)acrylate (*R*)-30b**

37% yield.

#### 3.1.9. General Procedure for the Preparation of 1-(Allyloxy)-1-oxo-3-phenylpropan-2-yl 2-Arylacrylic Acids

Allyl esters (14b–16b), morpholine and Pd(PPH_3_)_4_ are dissolved in anhydrous THF in a 1:20:0.1 stoichiometric ratio. Stirring occurred for 2 h at RT. The solvent is removed in vacuo and the residue is dissolved in CH_2_Cl_2_ and extracted with NaHCO_3ss_, the aqueous phase is then brought to acidic pH with HCl 2N and is finally extracted with CH_2_Cl_2_. The organic phase is washed with NaCl_ss_, dried with Na_2_SO_4_, filtered, and the solvent is removed in vacuo, affording a yellow oil which is further purified via column chromatography over silica gel. 

**(*R*)- and (*S*)-1-(allyloxy)-1-oxo-3-phenylpropan-2-yl-2-phenylacrylic acid 28b**

Column chromatography was performed using an eluent composed of Hex/EtOAc/Acetic acid (6.6:3.3:0.1) affording a transparent oil which was further purified forming the sodium salt of the desired product. 36–66% yield. **(*R*)-14b** [α]_D_^20^ = +57.2 (c = 2, MeOH). **(*S*)-14b** [α]_D_^20^ = −58.6 (c = 0.958, MeOH).

**1-(allyloxy)-1-oxo-3-(3,4-dimethoxyphenyl)-propan-2-yl-2-phenylacrylate 29b**

The crude oil is purified via the preparation of a cyclohexylamine salt without column chromatography. 84% yield. 

**(*R*)-1-(allyloxy)-1-oxo-3-phenyl-propan-2-yl 2-(3,4-dimethoxyphenyl)acrylate (*R*)-30b**

Column chromatography was conducted a CH_2_Cl_2_/MeOH 9:1 mixture as eluent, affording a transparent oil which was further purified forming the cyclohexylamine salt of the desired product.

20% yield. [α]_D_^20^ = +15.2 (c = 0.25; MeOH).

#### 3.1.10. Preparation of Methyl 2-Cinnamamido-3-phenylpropanoate **31a**

Cinnamic acid (2.07 mmol) is dissolved in anhydrous THF at 0 °C, to which L-phenylalanine methyl ester hydrochloride (1.86 mmol), triethylamine (1.67 mmol) in CHCl_3_, HOBt x H_2_O (1.43 mmol) in THF and DCC (1.78 mmol) in CHCl_3_ are added. The solution is stirred for 1 h at 0 °C, then at RT overnight. Afterwards, a precipitate is observed and filtered away; the solvent is evaporated away under vacuum and the residue is dissolved in EtOAc, washed once with distilled water, then once with a 10% aqueous solution of citric acid, once with NaHCO_3ss_, three times with NaOH 0.1N, and finally once with NaCl_ss_. The organic phase is dried over Na_2_SO_4_, filtered and the solvent is removed in vacuo, affording the desired product as a pale yellow solid. 27% yield.

#### 3.1.11. General Procedure for the Preparation of Methyl 2-(3-Arylacrylamido)-3-arylpropanoates **32a**, **34a**, **35a**

Appropriately substituted cinnamic acids (1 mmol) are dissolved in DMF; the system is cooled to 0 °C and then HOBt x H_2_O (1 mmol) and EDCI (1 mmol) are added. In a separate vessel, the appropriate L-aminoacid methyl ester hydrochloride (1 mmol) was mixed with N-methylmorpholine (1 mmol) in CH_2_Cl_2_ for 15 min, then this mixture is added to the main reaction vessel. Stirring occurred at RT overnight. The crude reaction mixture is diluted with NaHCO_3_ 5%, extracted five times with CH_2_Cl_2_, and the organic phase is washed once with NaCl_ss_, dried over anhydrous Na_2_SO_4_, filtered, and the solvent is evaporated in vacuo, affording a solid.

**Methyl 2-(3-(3,4-dimethoxyphenyl)acrylamido)-(*S*)-3-phenylpropanoate 32a**

The crude solid was purified via column chromatography over silica gel (eluent EtOAc/Hex 6:4), affording a white solid. 54% yield. 

**Methyl 2-cinnamamido-(*S*)-3-(4-hydroxyphenyl)propanoate 34a:**

The crude product did not require further purification and appeared as a white solid. 99% yield.

**Methyl 2- (3-(3,4-dimethoxyphenyl)acrylamido)-(*S*)-3-(4-hydroxyphenyl)propanoate 35a**

The crude solid is purified via column chromatography over silica gel using a EtOAc/Hex 6:4 mixture as eluent. The purified product appears as a white solid.

#### 3.1.12. Preparation of Methyl (*R*,*S*)-2-phenylpropanoylamido-(*S*)-3-phenylpropanoate **36a**

Briefly, 2-phenylpropanoic acid (2.32 mmol) is dissolved in DMF. EDCI (2.32 mmol) and HOBt x H_2_O (2.32 mmol) are added at 0 °C, while L-phenylalanine methyl ester hydrochloride (2.32 mmol) and N-methylmorpholine (2.32) are mixed in a separate vessel in CH_2_Cl_2_ for 10 min. The two mixtures are joined and stirred overnight at RT. The resulting solution is diluted with NaHCO_3_ 5%, extracted five times with CH_2_Cl_2_ and the organic phase is washed once with NaCl_ss_, dried over anhydrous Na_2_SO_4_, filtered and the solvent is evaporated away in vacuo. The residue, appearing as a yellow crude solid, is dissolved in EtOAc and washed with HCl 2N. Once the solvent is removed, the desired product is obtained as a white solid. 79% yield.

#### 3.1.13. Preparation of Methyl 2-(2-Naphthamido)-(*S*)-3-phenylpropanoate **37a**

2-naphthoic acid (1.16 mmol) is dissolved in DMF; the system is cooled to 0 °C, after which EDCI (1.16 mmol) and HOBt x H_2_O (1.16 mmol) are added. In a separate vessel, L-phenylalanine methyl ester hydrochloride (1.16 mmol) is mixed with N-methylmorpholine (1.16 mmol) in CH_2_Cl_2_ for 10 min. This mixture is then added to the naphtoic acid solution and the reaction is stirred overnight at RT. The resulting solution is diluted with NaHCO_3_ 5%, extracted 4 times with CH_2_Cl_2_ and the organic phase is washed once with NaCl_ss_, dried over anhydrous Na_2_SO_4_, filtered and the solvent is removed under vacuum. The residue is dissolved in EtOAc and washed with HCl 2N; the desired product appears as a pale yellow solid. 80% yield.

#### 3.1.14. General Procedure for the Preparation of 2-Substituted-3-arylpropanoic Acids **31**, **32**, **34**–**37**

The appropriate 2-substituted methyl 3-arylpropanoate (1 mmol) is dissolved in a THF/H_2_O 1:1 solution; the system is cooled to 0 °C and LiOH x H_2_O (5 mmol) is added. The mixture is stirred for 5 h at RT, then it is acidified with HCl 1N and extracted with EtOAc 4 times. The organic phase is separated, dried over anhydrous Na_2_SO_4_, filtered, and the solvent is removed under vacuum giving the crude product.

**2-cinnamamido-3-phenylpropanoic acid 31:**

The crude product appears as a transparent oil which is treated with Et_2_O/Hex 1:1 giving a white solid that is further purified via crystallization with Hex + EtOAc(gtt), affording the desired product as white crystals. 24% yield. mp: 194.2–198.2 °C

**(*S*)-2-(3-(3,4-dimethoxyphenyl)acrylamido)-3-phenylpropanoic acid 32:**

The crude product appears as an oil mixed with a white solid and is treated with Et_2_O/Hex 1:1 affording a white solid. 78.7% yield.

**(*S*)-2-cinnamamido-3-(4-hydroxyphenyl)propanoic acid 34:**

The crude product appears as a pale yellow oil which is treated with Et_2_O/Hex 1:1 affording a pale yellow solid. 76% yield.

**(*S*)-2-(3-(3,4-dimethoxyphenyl)acrylamido)-3-(4-hydroxyphenyl)propanoic acid 35:**

The crude product appears as a colorless oil and is treated with CHCl_3_/Hex 1:1 affording the desired product as a white powder. 96.6% yield. mp: 105.0–108.5 °C. [α]_D_^20^ = −29.66 (c = 1.00, MeOH).

**(*R,S*)-2-phenylpropanoylamido-(*S*)-3-phenylpropanoic acid 36:**

The crude product, a yellow solid, is crystallized in Et_2_O + Hex (gtt), affording the desired product as white crystals. 34% yield. mp: 66.5–69.3 °C

**(*S*)-2-(2-naphthamido)-3-phenylpropanoic acid 37:**

The crude product appears as a white solid that is further purified via crystallization at 5 °C in CHCl_3_ + Hex (gtt), affording white crystals. 70% yield. mp: 156.5–157.3 °C. [α]_D_^20^ = −84.61 (c = 1.001, MeOH).

#### 3.1.15. Preparation of (*S*)-2-(3-(3,4-Dihydroxyphenyl)acrylamido-3-phenylpropanoic Acid **33**

Briefly, 0.14 mmol of compound 32 are dissolved in CH_2_Cl_2_ and a 1 mL of a 1M solution of BBr_3_ is then added dropwise at room temperature. The reaction is stirred overnight at room temperature, after which it is quenched by adding H_2_O at −15 °C. The resulting mixture is extracted three times with EtOAc, dried over Na_2_SO_4_, filtered, and the solvent is evaporated under vacuum. The crude product is purified via column chromatography (eluent EtOAc:MeOH 7:3). 46% yield. m.p. 198–199 °C. [α]_D_^20^ = +6.465 (c = 1.00, MeOH).

### 3.2. Biological Methods

Reference compounds, cell culture mediums and other reagents, along with plates and other physical supports for cell culture and enzyme assays were purchased from Merck Sigma (Milan, Italy) and Invitrogen (Basel, Switzerland). The FAAH enzyme, the substrate AMC-AA and the reference compound JZL-195 for the FAAH inhibition assay were obtained from Cayman Chemical (Ann Arbor, MI, USA).

#### 3.2.1. Plasmids

The expression vectors carrying the chimeric receptors composed of the yeast GAL4-DNA binding domain fused to human PPARα, PPARγ or PPARδ, and their respective reporter plasmid (pGAL5TKpGL3), presenting the GAL4 response element in five repeats upstream of a minimal thymidine kinase promoter, itself upstream to the luciferase gene, were previously described [35]. These plasmids were kindly donated by Dr. Krister Bamberg (AstraZeneca, Mölndal, Sweden).

#### 3.2.2. Cell Culture and Transfection

HepG2 cell line (human hepatoblastoma, Interlab Cell Line Collection, Genoa, Italy) was cultured in Minimum Essential Medium (MEM) containing 10% fetal bovine serum, penicillin (100 U/mL) and streptomycin sulfate (100 µg/mL) at 37 °C in a humidified incubator in 5% CO_2_ atmosphere. Transactivation assays were conducted seeding 10^5^ cells/well in a 24-well plate. The cells were transfected after 24 h with the calcium phosphate method CAPHOS^®^, following the manufacturer’s guidelines. Plasmids encoding the fusion proteins GAL4-PPARa-LBD, GAL4-PPARg-LBD, GAL4-PPARd-LBD or GAL4-FXR-LBD (30 ng), pGAL5TKpGL3 (100 ng), and pCMVbgal (250 ng) were used. Treatment of the cells with the investigated compounds was carried out 4 h after transfection, in triplicate, and the cells were incubated in the treated medium for additional 20 h. Cells were then lysed and luciferase activity in the extract was determined via a VICTOR^3^ V Multilabel Plate Reader, PerkinElmer). 

#### 3.2.3. FAAH Inhibition Assay

Briefly, 96-well black flat-bottom microtiter NBS plates (COSTAR flat black) were utilized to perform the assays (in triplicate). The experiments in a total volume of 200 µL, first incubating different concentrations of each potential inhibitor in an appropriate fluorometric assay buffer (tris-HCl 125 mM, Na_2_EDTA · 2H_2_O 1 mM, pH = 9.0) with the enzyme (FAAH Human recombinant, Cayman Chemical, Ann Arbor, MI, USA) for 15 min at room temperature, keeping the plate in orbital shaking.

The substrate (7-amino-4-methyl-2H-1-benzopyran-2-one-5Z,8Z,11Z,14Z-eicosatetraen-amide, AMC-AA, 1 µM final concentration) was then added, and the assay was incubated for 2–3 h at 37 °C in a TECAN infinite M1000Pro plate reader (Tecan, Männedorf, Switzerland) which measured the fluorescence from each well every 30 s (λex = 340 nm, λem = 450 nm), determining FAAH activity as relative fluorescence units (RFU). Control wells lacking the inhibitor and blank wells lacking both inhibitor and enzyme were used to calculate percent inhibition for each tested compound. IC_50_ values were calculated via GraphPad Prism 5.0 (GraphPad Software, La Jolla, CA, USA) and are reported as mean ± SEM of at least two independent measurements performed in triplicate.

### 3.3. Molecular Modeling

Compounds **5**, **14,** and **18** were built using the Maestro software package (Schrödinger Release 2020-2, Maestro, Schrödinger, LLC, New York, NY, USA, 2019), and the relative ionization state was set to its most energetically favorable one for pH = 7.4 by the FixpKa suite of QUACPAC [36]. The published X-ray data (pdb ID 4DO3) [26] was used as biomolecular target. Chain A of the enzyme structure was passed to the Protein Preparation Wizard interface of MAESTRO for removing water molecules, and hydrogen atoms added, optimizing their position, and determining the protonation states of residues according to PROPKA prediction at pH 7.0. AMBER UNITED force field electrostatic charges [37] were loaded on the protein structure, while the molcharge complement of QUACPAC was used to achieve Marsili-Gasteiger charges for the inhibitors. Binding mode of each derivative was sampled with a thousand runs of Lamarckian Genetic Algorithm (LGA) implemented in AUTODOCK 4.2.6 [38] using the GPU-OpenCL algorithm version [39]. To accomplish this task, affinity maps were first calculated on a 85∙85∙85 Å^3^ box, 0.375 Å spaced, centered on the co-crystallized carprofen, and LGA runs were issued with population size and the number of energy evaluations to 300 and 10,000,000, respectively, considering water contribution according to the hydration force field of AUTODOCK [40]. All the docked poses were thereafter clustered, and the best free energy/most populated pose selected as representative of the binder’s mode.

## 4. Conclusions

A panel of compounds comprising natural compounds and synthetic PPAR agonists was tested for FAAH inhibition. As a result, a few aryloxyacetic derivatives were found to be active as FAAH inhibitors and PPAR agonists. In particular, a lack of steric bulk on the alpha carbon of the acetic moiety, and an aromatic moiety composed of at least two phenyl rings connected by a short linker are favored for PPAR-FAAH dual activity. However, a trans-stilbenic moiety seems to recover FAAH activity even if a bulky group is present on the alpha carbon, as in the case of compound **18**, producing a more balanced activity profile. Indeed, compound **18** is the first known compound able to achieve the desired activity profile with sufficient potency towards all targets.

Docking studies performed on some selected aryloxyacetic FAAH inhibitors, along with the experimental data that have been collected, allowed to rationalize their inhibition activity, suggesting that the development of novel compounds, with a lipophilic, flat and rigid aromatic tail and with a balanced overall lipophilicity could yield the desired activity profile. Although the binding site of these carboxylic inhibitors of FAAH does not show stereoselectivity, it is important to note that their activity as PPAR agonists, as shown in the previously reported data, often does (with a preference for the (*S*) configuration for simple aryloxyacetic acids and for the (*R*) configuration for ureidofibrates). 

This study stands as proof that it is indeed possible to have compounds acting on both FAAH and PPARs. Further developments along this line of research could lead to a better understanding of the workings and the connections between the endocannabinoid system and the nuclear receptors such as PPARs, and to the discovery of novel compounds with many possible therapeutic applications.

## Figures and Tables

**Figure 2:**
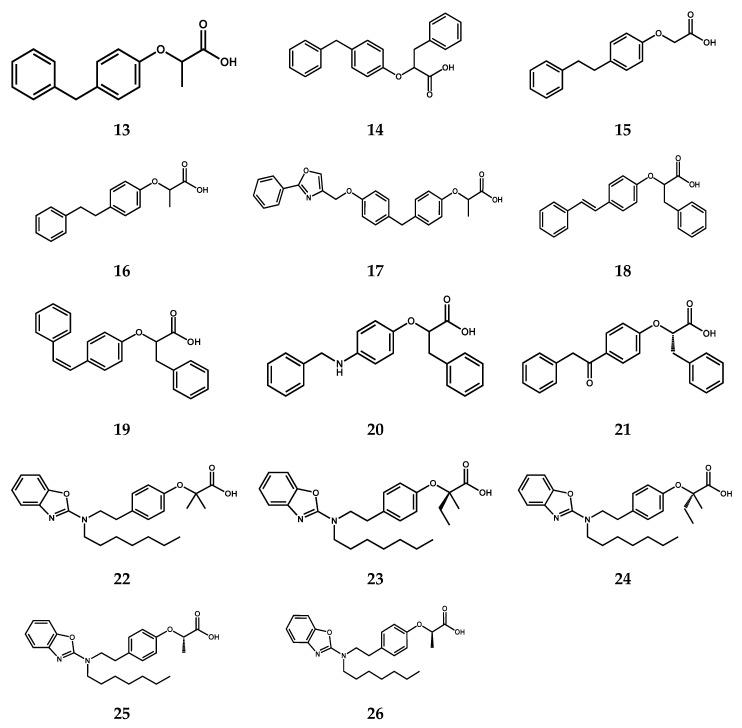
Molecular structures of phenoxyacetic analogues.

**Table 1:** Biological activities of PPAR standards **1**–**3** and the four most promising compounds from the initial set. i: inactive.

Compound	FAAH	PPARα		PPARγ	
	IC_50_ (μM)	EC_50_ (µM)	E_max_	EC_50_ (µM)	E_max_
**1**	i	i		0.04 ± 0.02	100 ± 9
**2**	i	1.6 ± 0.3	100 ± 10	i	
**3**	>100 (40 ± 20% @ 100 μM)	i		i	
**4**	≈100 (62.05 ± 5.00% @ 100 μM)	i		i	
**5**	40.2 ± 17.7	24 ± 5	34 ± 8%	33 ± 23	9 ± 6%
**8** [29]	30.5 ± 12.6	0.12 ± 0.16	87 ± 7%	0.83 ± 0.75	37 ± 11%
**12**	26.1 ± 11.4	i		i	
**JZL195**	0.019 ± 0.003	-		-	

To shed more light on the structure–activity relationships of phenoxyacetic acids towards FAAH, we decided to add to the screening a new series of analogues, some of which are previously unpublished (Figure 2).

**Table 2:** Biological activities of phenoxyacetic compounds **13**–**26**. i: inactive.

Compound	FAAH	PPARα		PPARγ	
	IC_50_ (μM)	EC_50_ (µM)	E_max_	EC_50_ (µM)	E_max_
**13**	>100 (30.0 ± 3.0% @ 100 μM)	4.6 ± 1.8	116 ± 1%	i	
**14** [31]	i	0.0159 ± 0.0003	115 ± 27%	0.58 ± 0.19	42 ± 1%
**15**	≈100 (63 ± 14% @ 100 μM)	i		i	
**16**	>100 (32.2 ± 2.8% @100 μM)	17.6 ± 3	116 ± 1%	24 ± 6	18 ± 7%
**17** [32]	i	0.039 ± 0.013	47 ± 13	0.29 ± 0.08	26 ± 3
**18** [31]	24 ± 2.5	1.75 ± 0.12	57 ± 4%	0.72 ± 0.27	50 ± 1%
**19** [31]	>100 (30.5 ± 3.2% @ 100 μM)	0.16 ± 0.02	107 ± 2%	1.5 ± 0.4	34 ± 1%
**20** [31]	>100 (39.2 ± 7.3% @ 100 μM)	3.6 ± 1.5	78 ± 4%	13 ± 3	27 ± 6%
**21** [31]	>100 (34.0 ± 4.6% @ 100 μM)	1.8 ± 1.0	106 ± 1%	9.1 ± 0.9	40 ± 2%
**22** [29]	>100 (9.9 ± 3.8% @ 100 μM)	0.030 ± 0.016	82 ± 7%	0.11 ± 0.06	50 ± 4%
**23** [29]	>100 (12.9 ± 3.3% @ 100 μM)	0.003 ± 0.001	91 ± 3%	0.07 ± 0.05	116 ± 9%
**24** [29]	>100 (8.7 ± 1.8% @ 100 μM)	0.056 ± 0.034	75 ± 4%	0.59 ± 0.11	50 ± 5%
**25** [29]	≈100 (45.1 ± 7.3% @ 100 μM)	0.62 ± 0.26	78 ± 4%	2.3 ± 1.0	32 ± 5%
**26** [29]	>100 (35 ± 12% @ 100 μM)	0.025 ± 0.017	93 ± 6%	0.15 ± 0.06	59 ± 2%

**Table 3:**
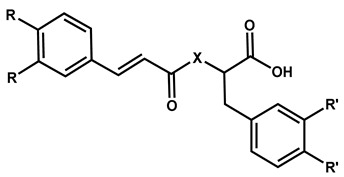
Molecular structures of tested derivatives of rosmarinic acid.

Compound	R	R’	X
***27* (*Clovamide*)**	OH	OH	NH
**(*R*)-28, (*S*)-28**	H	H	O
**29**	OCH_3_	H	O
**(*R*)-30**	H	OCH_3_	O
**31**	H	H	NH
**32**	OCH_3_	H	NH
**33**	OH	H	NH
**34**	H	4-OH	NH
**35**	OCH_3_	4-OH	NH
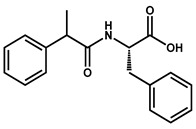		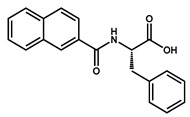	
**36**		**37**	

**Table 4:** Biological activities of the only active derivatives of rosmarinic acid. i: inactive.

Compound	FAAH	PPARα		PPARγ	
	IC_50_ (μM)	EC_50_ (µM)	E_max_	EC_50_ (µM)	E_max_
***(S)*-28**	i	24 ± 5	34 ± 8%	33 ± 23	9 ± 6%
**33**	12.6 ± 3.1	i		i	

**Table 5:** ^(a)^ Free energy of binding (kcal/mol) as estimated by the AutoDock scoring function. ^(b)^ Ligand efficacy measured as the absolute FEB value divided by the number of heavy atoms. ^(c)^ Frequency of the selected cluster. ^(d)^ Contact surface area in Å^2^.

Compound	FEB ^a^	LE ^b^	POP ^c^	CSA ^d^
***5***	−7.10	−0.395	45/1000	351.02
**(*R*)-14**	−8.97	−0.359	258/1000	385.37
**(*S*)-14**	−8.96	−0.358	131/1000	436.09
**(*R*)-18**	−9.29	−0.357	256/1000	484.92
**(*S*)-18**	−9.37	−0.360	170/1000	482.02

## Supplementary Materials

Supplementary Materials can be found at https://www.mdpi.com/1422-0067/21/19/7026/s1. Spectral analysis data; Figure S1: Docking pose of compound **8** into the FAAH binding site (Free energy of binding = −8.53 kcal/mol; Ligand efficacy = −0.280; Frequency of the selected cluster = 58/1000).

## Abbreviations

AEA	Arachidonoylethanolamide, Anandamide
CNS	Central Nervous System
COX	Cyclooxigenase
ECS	Endocannabinoid System
ER	Endoplasmic Reticulum
GC-MS	Gas Chromatography—Mass Spectrometry
HRMS	High Resolution Mass Spectrometry
FAAH	Fatty Acid Amide Hydrolase
NAAA	N-Acylethanolamine Acid Amidase
NAEs	N-Acylethanolamides
OEA	Oleoylethanolamide
PEA	Palmitoylethanolamide
PPARs	Peroxisome Proliferator-Activated Receptors
NMR	Nuclear Magnetic Resonance
T2DM	Type 2 Diabetes Mellitus
