# Supplementary material for: Beyond the Canonical Endocannabinoid System. A Screening of PPAR Ligands as FAAH Inhibitors
Source: Int J Mol Sci. 2020 Sep 24;21(19):7026. doi: 10.3390/ijms21197026 (PMC7582602; doi:10.3390/ijms21197026)
Supplement: Supplementary file 1 [file ijms-21-07026-s001.pdf]

Supplementary Information

Beyond the canonical Endocannabinoid System. A screening of PPAR ligands as FAAH inhibitors.

Leonardo Brunetti[1], Antonio Carrieri[1], Luca Piemontese[1], Paolo Tortorella[1], Fulvio Loiodice[1,*], Antonio Laghezza [1,*].

[1] Department of Pharmacy and Pharmaceutical Sciences, University of Bari "A. Moro", via E. Orabona 4, 70125, Bari, Italy.

* Correspondence: fulvio.loiodice@uniba.it (F.L.), antonio.laghezza@uniba.it (A.L.).

1. Spectral analysis data

1.1 Ethyl 4-benzyl-phenoxyacetate (5a)

GC-MS: m/z (%): 270 (99, M+); 183 (100).
^{1}H-NMR ($CDCl_3$, 300MHz): δ = 1.29 (t, 3H, CH_3CH_2), 3.92 (s, 2H, $PhCH_2Ph$), 4.26 (q, 2H, CH_3CH_2), 4.59 (s, 2H, OCH_2COOEt), 6.82-7.30 (m, 9H, aromatic).

1.2 (R,S)-Ethyl 2-(4-benzylphenoxy)-propanoate (13a)

GC-MS: m/z (%): 284 (98, M+); 211 (100), 183 (30), 91 (21).
^{1}H-NMR ($CDCl_3$, 300MHz): δ = 1.24 (t, 3H, CH_3-CH_2), 1.60 (d, 3H, CH_3CH), 3.91 (s, 2H, $PhCH_2Ph$), 4.21 (q, 2H, CH_2CH_3), 4.70 (q, 1H, $CHCH_3$), 6.77-7.31 (m, 9H, aromatic).

1.3 Ethyl trans-(4-styryl-phenoxy)-acetate (15a)

GC-MS: m/z (%): 282 (100, M+), 195 (35), 178 (36).
^{1}H-NMR ($CDCl_3$, 300MHz): δ = 1.31 (t, 3H, CH_3CH_2), 4.28 (q, 2H, CH_2CH_3), 4.64 (s, 2H, OCH_2COOEt), 6.88-6.95 (m, 2H, aromatic), 6.98 (d, 1H, CH=*CH*), δ 7.07 (d, 1H, *CH*=CH), δ 7.21-7.50 (m, 7H, aromatic).

1.4 (R,S)-Ethyl 2-trans-(4-styryl-phenoxy)-propanoate (16a)

GC-MS: m/z (%): 296 (100, M+), 195 (65), 178 (28).
^{1}H-NMR ($CDCl_3$, 300MHz): δ = 1.26 (t, 3H, CH_3CH_2), 1.63 (d, 3H, $CHCH_3$), 4.23 (q, 2H, CH_2CH_3), 4.77 (q, 1H, $CHCH_3$), 6.85-6.88 (m, 2H, aromatic), 6.98 (d, 1H, CH=*CH*), 7.05 (d, 1H , *CH*=CH), 7.21-7.49 (m, 7H, aromatic).

1.5 Ethyl (4-phenethyl-phenoxy)-acetate (15b)

GC-MS: m/z (%): 284 (15, M+), 193 (100), 107 (11).
^{1}H-NMR ($CDCl_3$, 300MHz): δ = 1.30 (t, 3H, CH3CH2), 2.84-2.90 (m, 4H, PhCH2CH2Ph), 4.27 (q, 2H,CH2CH3), 4.60 (s, 2H, OCH2COOEt), 6.80-7.30 (m, 9H, aromatic).

1.6 Ethyl 2-(4-phenethyl-phenoxy)-propanoate (16b)

GC-MS: m/z (%): 298 (M+, 18), 207 (100), 107 (60).
^{1}H-NMR ($CDCl_3$, 300MHz): δ = 1.25 (t, 3H, CH_3CH_2), 1.60 (d, 3H, CH_3CH), 2.84-2.88 (m, 4H, $PhCH_2CH_2Ph$), 4.22 (q, 2H, CH_2CH_3), 4.71 (q, 1H, $CHCH_3$), 6,78-7,30 (m, 9H, aromatic).

1.7 4-benzyl-phenoxyacetic acid (5)

GC-MS (after esterification with diazomethane): m/z (%): 256 (M+, 89), 183 (100), 165 (50), 152 (28).

^{1}H-NMR ($CDCl_3$, 300MHz): δ = 3.93 (s, 2H, Ph*CH*$_2$Ph), 4.64 (s, 2H, O*CH*$_2$COOH), 6.83-7.31 (m, 9H, aromatic).

1.8. (R,S)-2-(4-benzyl)-phenoxypropanoic acid 13

GC-MS (after esterification with diazomethane): m/z (%): 270 (M+, 100), 211 (77), 183 (41).

^{1}H-NMR ($CDCl_3$, 300MHz): δ = 1.16 (d, 3H,*CH*$_3$CH), 3.92 (s, 2H, Ph*CH*$_2$Ph); 4.75 (q, 1H, O*CH*), 6.79-7.31 (m, 9H, aromatic).

1.9. 4-phenethyl-phenoxyacetic acid 15

GC-MS (after esterification with diazomethane): m/z (%): 270 (M+, 12), 179 (100).

^{1}H-NMR ($CDCl_3$, 300MHz): δ = 2.81-2.89 (m, 4H, Ph*CH*$_2$*CH*$_2$Ph), 4.66 (s, 2H, O*CH*$_2$), 6.81-7.30 (m, 9H, aromatic).

1.10. (R,S)-2-(4-phenethyl)-phenoxyacetic acid 16

GC-MS (after esterification with diazomethane): m/z (%): 284 (M+, 1), 193 (100), 107 (84).

^{1}H-NMR ($CDCl_3$, 300MHz): δ = 1.64 (d, 3H, *CH*$_3$CH), 2.83-2.90 (m, 4H, Ph*CH*$_2$*CH*$_2$-Ph), 4.76 (q, 1H, *CH*CH_3), 6.79-7.30 (m,9H, aromatic).

1.11. 4-[(3,4-dimethoxyphenyl)methyl-idene]-2-methyl-4,5-dihydro-1,3-oxazol-5-one (29c)

GC-MS: m/z (%): 247 (35, M+), 177 (100), 162 (34).

1.12. 3-(3,4-dimethoxyphenyl)-2-hydroxyacrylic acid (29d)

^{1}H-NMR($CDCl_3$, 500MHz): δ = 3.87 (s, 1H, CHO*H*), 3.91 (s, 3H, OCH_3), 3.92 (s, 3H, OCH_3), 4.15 (s, 1H, CH), 6.65 (s, 1H, COOH), 6.88 (s, 1H, aromatic), 7.29 (s, 1H, aromatic), 7.50 (s, 1H, aromatic).

1.13. (R,S)-3-(3,4-dimethoxyphenyl)-2-hydroxypropanoic acid (29e)

HRMS: $[M-H]^-$ calculated 225.0768; found 225.0765.

1.14. (R)- and (S)-Allyl 3-Phenyl-2-hydroxypropanoate (28a)

GC-MS: m/z (%): 206 (1, M+), 188 (15), 91 (100); H^1-NMR ($CDCl_3$, 500MHz): δ = 3.00 (dd, 1H, CH_2, J: 14.19, 6.85, 4.40 Hz), 3.15 (dd, 1H, CH_2 J:14.19, 6.85 Hz) 4.49 (q, 1H, CH, J: 6.85, 4.40 Hz), 4.67 (d, 2H, CH_2, J: 5.87 Hz), 5.35 (dd, 2H, CH_2, J: 0.98, 16.4 Hz), 5.87-5.95 (m, 1H, CH), 7.23-7.32 (m, 5H, aromatic).

1.15. Allyl 3-(3,4-dimethoxyphenyl)-2-hydroxypropanoate (29a)

GC-MS: m/z%= 266 (19, M+), 151 (100); ^{1}H-NMR($CDCl_3$, 500MHz): δ = 2.71 (d, 1H, OH), 2.93 (dd, 1H, CH_2, J: 14.19, 6.36 Hz), 3.09 (dd, 1H, CH_2, J: 14.19, 4.40 Hz), 3.85 (s, 6H, $(OCH_3)_2$), 4.45 (dd, 2H, CH=*CH*$_2$, J: 6.36, 4.40, 1.96 Hz), 4.65 (dt, 2H, CH_2, J: 5.87 Hz), 5.32 (d, 2H, CH_2, J: 11.74 Hz), 5.91 (m, 1H, CH), 6.74-76 (m, 1H, aromatic), 6.78 (s, 1H, aromatic), 6.79 (s, 1H, aromatic).

1.16. (R)- and (S)-1-(allyloxy)-1-oxo-3-phenylpropan-2-yl 2-phenylacrylate ((R)-28b)

GC-MS: m/z (%)= 336 (1, M+), 188 (27), 149 (24), 131 (100), 103 (30); ^{1}H-NMR ($CDCl_3$, 500MHz): δ = 3.19-3.29 (m, 2H, CH_2), 4.65 (d, 2H, CH_2), 5.28 (dd, 2H, CH*CH*$_2$), 5.38 (dd, 1H, CH, J: 4.4Hz), 5.87-5.95 (m, 1H, CH), 6.47 (d, 1H, PhCHC*H*, J: 15.65 Hz), 7.24-7.33 (m, 5H, aromatic), 7.40 (t, 3H, aromatic), 7.53 (dd, 2H, aromatic), 7.70 (d, 1H, PhC*H*CH, J: 15.65).

1.17. 1-(allyloxy)-1-oxo-3-(3,4-dimethoxyphenyl)-propan-2-yl 2-phenylacrylate (29b)

GC-MS: m/z (%) = 396 (23, M+), 208 (100), 191 (63).

1.18. (R)-1-(allyloxy)-1-oxo-3-phenyl-propan-2-yl 2-(3,4-dimethoxyphenyl)-acrylate ((R)-30b)

^{1}H-NMR ($CDCl_3$, 500MHz): 3.24 (dd, 2H, CH_2, J: 14.18 Hz), 3.92 (s, 6H, $(OCH_3)_2$), 4.64 (d, 2H, CH_2, J: 11.25 Hz), 5.28 (d, 2H, CH_2CHCH_2, J: 5.87 Hz), 5.40 (t, 1H, CH, J: 14.18 Hz), 5.87 (m, 1H, CH_2CHCH_2), 6.34 (d, 1H, PhCHC*H*, J: 15.65), 6.87 (m, 1H, aromatic), 7.05 (m, 1H, aromatic), 7.10 (m, 1H, aromatic), 7.21-7.32 (m, 5H, aromatic), 7.65 (d, 1H, PhC*H*CH, J: 15.65).

1.19. (R)- and (S)-1-(allyloxy)-1-oxo-3-phenylpropan-2-yl 2-phenylacrylic acid (28)

HRMS: $[M-H]^-$ calculated 295.0976; found: 295.0972; ^{1}H-NMR ($CDCl_3$, 500MHz): δ = 3.21-3.33 (m 2H, CH_2), 5.40 (dd, 2H, CH_2, J: 3.91 Hz), 6.46 (d, 1H, PhCHC*H*, J: 15.65), 7.25-7.35 (m, 5H, aromatic), 7.37-7.41 (m, 3H, aromatic), 7.53 (m, 2H, aromatic), 7.70 (d, 1H, PhC*H*CH, J: 15.65). **(*R*)-14b** $[\alpha]_D^{20}$ = +57.2° (c=2, MeOH). **(*S*)-14b** $[\alpha]_D^{20}$ = -58.6° (c=0.958, MeOH).

1.20. 1-(allyloxy)-1-oxo-3-(3,4-dimethoxyphenyl)-propan-2-yl 2-phenylacrylic acid (29)

^{1}H-NMR ($CDCl_3$, 500MHz): δ = 1.11-1.56 (m, 6H, $(CH_2)_3$), 1.69 (d, 2H, CH_2), 1.98 (d, 2H, CH_2), 2.89 (m, 1H, CH), 3.08 (dd, 1H, CH_2, J: 10.27, 14.19 Hz), 3.23 (dd, 1H, CH_2, J: 14.19 Hz), 3.81 (s, 3H, OCH_3), 3.82 (s, 3H, OCH_3), 5.09 (dd, 1H, CH, J: 10.27, 14.19 Hz), 6.41 (d, 1H, PhCHCH, J: 15.65), 6.75-6.83 (m, 3H, aromatic), 7.32-7.40 (m, 5H, aromatic), 7.58 (d, 1H, PhC*H*CH, J: 15.65).

1.21. (R)-1-(allyloxy)-1-oxo-3-phenyl-propan-2-yl 2-(3,4-dimethoxyphenyl)-acrylic acid ((R)-30b)

^{1}H-NMR ($CDCl_3$, 500MHz): δ = 1.12-1.55 (m, 6H, $(CH_2)_3$), 1.68 (d, 2H, CH_2), 1.96 (d, 2H, CH_2), 2.83-2.90 (m, 1H, CH), 3.13 (dd, 1H, CH_2, J: 24.40 Hz), 3.28 (dd, 1H, CH_2, J: 5.86, 24.40 Hz), 3.84 (s, 3H, OCH_3), 3.88 (s, 3H, OCH_3), 5.13 (dd, 1H, CH, J: 5.86, 24.40 Hz), 6.30 (d, 1H, PhCHC*H*, J: 26.36 Hz), 6.80 (s, 1H, aromatic), 6.97 (s, 2H, aromatic), 7.20-7.32 (m, 5H, aromatic), 7.53 (d, 1H, PhC*H*CH, J:26.36 Hz). $[\alpha]_D^{20}$ = +15.2° (c = 0.25; MeOH).

1.22. Preparation of methyl 2-cinnamamido-3-phenylpropanoate (31a)

GC-MS: m/z (%): 309 (6, M+), 146 (22), 131 (100), 103 (31). ^{1}H-NMR ($CDCl_3$, 500MHz): δ = 3.22 (m, 2H, CH_2), 3.76 (s, 3H, CH_3), 5.04 (m, 1H, CH), 6.08 (d, 1H, NH, J: 7.83 Hz), 6.40 (d, 1H, CH, J:15.7 Hz), 7.11-7.51 (m, 10H, aromatic), 7.64 (d, 1H, CH, J: 15.7 Hz).

1.23. Methyl 2-(3-(3,4-dimethoxyphenyl)acrylamido)-(S)-3-phenylpropanoate (32a)

GC-MS: m/z (%): 369 (13, M+), 206 (37), 191 (100), 91 (13.7).

1.24. Methyl 2-cinnamamido-(S)-3-(4-hydroxyphenyl)-propanoate (34a)

GC-MS: m/z (%): 325 (22, M+), 178 (56), 131 (100), 103 (48), 77 (30).

1.25. Methyl 2- (3-(3,4-dimethoxyphenyl)acrylamido)-(S)-3-(4-hydroxyphenyl)-propanoate (35a)

GC-MS: m/z (%): 385 (12, M+), 206 (78), 191 (100). ^{1}H-NMR ($CDCl_3$, 500MHz): δ = 3.10-3.15 (m, 2H, CH_2), 3.75 (s, 3H, OCH_3), 3.88 (s, 6H, $(OCH_3)_2$), 4.98-5.00 (m, 1H, CH), 6.07 (d, 1H, NH, J: 6.60 Hz), 6.27 (d, 1H, CH, J: 15.6), 6.74-7.26 (m, 7H, aromatic), 7.56 (d, 1H, CH, J: 15.6).

1.26. Methyl (R,S)-2-phenylpropanoylamido-(S)-3-phenylpropanoate (36a)

GC-MS: m/z (%): 311 (3, M+), 178 (18), 105 (100). ^{1}H-NMR ($CDCl_3$, 500MHz): δ = 1.47-1.51 (dd, 3H, CH3, J: 7.34 Hz, 16.14 Hz), 2.95-3.11 (m, 2H, CH_2), 3.50-3.58 (dq, 1H, CH, J: 7.34 Hz, 15.65 Hz), 3.67-3.70 (d, 3H, OCH_3, J: 16.14 Hz), 4.77-4.89 (dq, 1H, CH, J: 5.87 Hz, 37.18 Hz), 5.73-5.78 (m, 1H, NH), 6.72-6.73 (d, 1H, aromatic, J: 7.34 Hz), 6.92 (s, 1H, aromatic), 7.09-7.17 (m, 2H, aromatic), 7.21-7.34 (m, 7H, aromatic).

1.27. Methyl 2-(2-naphtamido)-(S)-3-phenylpropanoate (37a)

GC-MS: m/z (%): 333 (40, M+), 155 (100), 127 (63). ^{1}H-NMR (($(CD_3)_2CO$, 500MHz): δ = 3.19-3.33 (m, 2H, CH_2), 3.71 (s, 3H, CH_3), 4.94-4.99 (m, 1H, CH), 7.20-8.41 (m, 13H, aromatics + NH).

1.28. 2-cinnamamido-3-phenylpropanoic acid (31)

^{1}H-NMR (DMSO-d_6, 500MHz): δ = 2.89-3.13 (m, 2H, CH_2), 4.52-4.56 (m, 1H, CH), 6.69 (d, 1H, CH, J: 15.70 Hz), 7.16-7.53 (m, 11H, aromatic + CH), 8.35 (d, 1H, NH, J: 7.83 Hz).

1.29. (S)-2-(3-(3,4-dimethoxyphenyl)-acrylamido)-3-phenylpropanoic acid (32)

^{1}H-NMR ($CDCl_3$, 500MHz): δ = 3.13-3.36 (m, 2H, CH_2), 3.89 (s, 6H, $(OCH_3)_2$), 4.96-5.03 (m, 1H, CH), 5.19-5.65 (s, 1H, OH), 6.07 (d, 1H, NH, J: 7.03), 6.24 (d, 1H, CH, J: 15.50), 6.82-7.31 (m, 8H, aromatic), 7.58 (d, 1H, CH, J: 15.50).

1.30. (S)-2-cinnamamido-3-(4-hydroxyphenyl)-propanoic acid (34)

HRMS: $[2M-H]^-$ calculated: 621.2242, found: 621.2233

1.31. (S)-2-(3-(3,4-dimethoxyphenyl)-acrylamido)-3-(4-hydroxyphenyl)-propanoic acid (35)

HRMS: $[2M-H]^-$ calculated: 741.2665, found: 741.2660 (100.0). ^{1}H-NMR (DMSO-d_6, 300MHz): δ = 2.72-3.00 (m, 2H, CH_2), 3.77 (s, 3H, OCH_3), 3.78 (s, 3H, OCH_3), 4.42-4.47 (m, 1H, CH), 6.56-6.65 (m, 3H, aromatic), 6.95-7.13 (m, 5H, aromatic + CH), 7.30 (d, 1H, CH, J: 15.80 Hz), 8.15 (d, 1H, NH, J: 7.61 Hz).

1.32. (R,S)-2-phenylpropanoylamido-(S)-3-phenylpropanoic acid (36)

HRMS: $[M-H]^-$ calculated: 296.1292, found: 296.1291. ^{1}H-NMR ($CDCl_3$, 300MHz): δ = 1.45-1.51 (dd, 3H, CH_3, J: 7.20 Hz, 10.27 Hz), 2.96-3.18 (m, 2H, CH_2), 3.49-3.60 (m, 1H, CH), 4.72-4.87 (dq, 1H, CH, J: 5.56 Hz, 27.45 Hz), 4.91-5.63 (wide, 1H, COOH), 5.73-5.77 (m, 1H, NH), 6.78-6.81 (m, 1H, aromatic), 6.90-6.98 (m, 1H, aromatic), 7.09-7.35 (m, 8H, aromatic).

1.33. (S)-2-(2-naphtamido)-3-phenylpropanoic acid (37)

HRMS: $[M-H]^-$ calculated: 318.1136, found: 318.1134. ^{1}H-NMR ($CDCl_3$, 500MHz): δ = 3.30-3.45 (m, 2H, CH_2), 5.13-5.17 (m, 1H, CH), 6.73 (d, 1H, NH, J: 6.85 Hz), 7.23-8.20 (m, 12H, aromatic). mp: 156.5-157.3°C. $[\alpha]_D^{20}$ = -84.61° (c = 1.001, MeOH).

1.34 (S)-2-(3-(3,4-dihydroxyphenyl)-acrylamido)-3-phenylpropanoic acid (33)

HRMS: $[M-H]^-$ calculated: 326.1034, found: 326.1035. ^{1}H-NMR (DMSO-d_6, 500MHz): δ = 2.90-2.93 (m, 1H, CH), 3.11-3.14 (m, 2H, CH_2), 6.45 (d, J: 15.7 Hz, 1H, NH), 6.70 (d, J: 8.08 Hz, 1H, vinyl CH), 6.80 (d, J: 8.08 Hz, 1H, vinyl CH), 7.10-7.19 (m, 7H, aromatic), 7.58 (s, 1H, OH), 9.41 (s, 2H, $(OH)_2$).

2. Additional figures

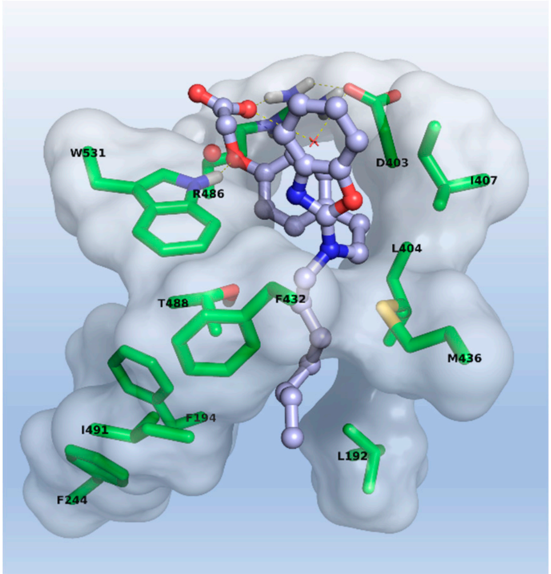

Figure S1. Docking pose of compound **8** into the FAAH binding site (Free energy of binding = -8.53 kcal/mol; Ligand efficacy = -0.280; Frequency of the selected cluster = 58/1000)
